# Supplementary material for: Limb, joint and pelvic kinematic control in the quail coping with steps upwards and downwards
Source: Sci Rep. 2022 Sep 23;12:15901. doi: 10.1038/s41598-022-20247-y (PMC9508109; doi:10.1038/s41598-022-20247-y)
Supplement: Supplementary file 1 — Supplementary Information. [file 41598_2022_20247_MOESM1_ESM.docx]

**Supplementary information:**

**Limb, joint and pelvic kinematic control in the quail coping with steps upwards and downwards**

**Emanuel Andrada ^(1)^, Oliver Mothes ^(2)^, Heiko Stark ^(1)^, Matthew C. Tresch ^(3)^, Joachim Denzler ^(2)^, Martin S. Fischer ^(1)^, Reinhard Blickhan ^(4)^**

1. Institute of Zoology and Evolutionary Research, Friedrich-Schiller-University Jena, Germany

2. Computer Vision Group, Friedrich-Schiller-University Jena, Germany

3. Department of Physiology, Northwestern University, Chicago, IL, USA

4. Science of Motion, Friedrich-Schiller-University Jena, Germany.

Corresponding author:

Emanuel Andrada (emanuel.andrada@uni-jena.de)

Keywords: avian locomotion, leg kinematics, uneven locomotion, 3D-locomotion

Table S1. Mean, median, max, min values and multiple comparisons for the effective leg during level and step locomotion. For the trailing limb, analyses were performed at early stance (15% of the stride ± 4%). For the leading limb, around TD (TD ± 4%).

|  |  | leg | step up | | | step down | | | level |
| --- | --- | --- | --- | --- | --- | --- | --- | --- | --- |
|  |  |  | 1 cm | 2.5 cm | 5 cm | 1 cm | 2.5 cm | 5 cm |  |
| leg length [m] | n | tr | 51 | 62 | 82 | 11 | 89 | 58 | 249 |
|  |  | le | 90 | 138 | 144 | 18 | 108 | 81 | 132 |
|  | mean +/- sd | tr | 0.123 +/- 0.007 | 0.128 +/- 0.004 | 0.132 +/- 0.01 | 0.115 +/- 0.016 | 0.132 +/- 0.005 | 0.119 +/- 0.006 | 0.11 +/- 0.008 |
|  |  | le | 0.145 +/- 0.007 | 0.144 +/- 0.008 | 0.142 +/- 0.009 | 0.146 +/- 0.004 | 0.148 +/- 0.007 | 0.137 +/- 0.007 | 0.128 +/- 0.007 |
|  | median | tr | 0.124 | 0.127 | 0.135 | 0.12 | 0.132 | 0.119 | 0.109 |
|  |  | le | 0.145 | 0.146 | 0.144 | 0.146 | 0.147 | 0.137 | 0.128 |
|  | max | tr | 0.136 | 0.138 | 0.145 | 0.13 | 0.143 | 0.135 | 0.137 |
|  |  | le | 0.156 | 0.156 | 0.156 | 0.157 | 0.17 | 0.151 | 0.147 |
|  | min | tr | 0.105 | 0.118 | 0.105 | 0.083 | 0.116 | 0.11 | 0.091 |
|  |  | le | 0.129 | 0.123 | 0.112 | 0.139 | 0.135 | 0.12 | 0.111 |
|  | comp | tr | 1 vs lev (****) | 2.5 vs lev (****)  2.5 vs 1(*) | 5 vs lev (****)  5 vs 2.5 (*)  5 vs 1 (****) | 1 vs lev (n.s.) | 2.5 vs lev (****)  2.5 vs 1 (****) | 5 vs lev (****)  5 vs 2.5 (****)  5 vs 1 (n.s.) |  |
|  |  | le | 1 vs lev (****) | 2.5 vs lev (****)  2.5 vs 1 (n.s.) | 5 vs lev (****)  5 vs 2.5 (n.s.)  5 vs 1 (n.s.) | 1 vs lev (****) | 2.5 vs lev (****)  2.5 vs 1 (n.s.) | 5 vs lev (****)  5 vs 2.5 (****)  5 vs 1 (****) |  |
| Leg angle at TD (α_0_) [°] | n | tr | 51 | 53 | 82 | 24 | 107 | 58 | 249 |
|  |  | le | 90 | 129 | 144 | 18 | 126 | 81 | 132 |
|  | mean +/- sd | tr | 53.5 +/- 3.1 | 56.9 +/- 4 | 63.1 +/- 4.2 | 52.1 +/- 8.5 | 53 +/- 3.2 | 52 +/- 6.2 | 54.3 +/- 3.9 |
|  |  | le | 37.8 +/- 4.8 | 39 +/- 4.5 | 35.7 +/- 5.2 | 50.4 +/- 7 | 54.5 +/- 5.5 | 53 +/- 3.9 | 42.4 +/- 3.9 |
|  | median | tr | 53.8 | 57.3 | 62.7 | 55.3 | 52.6 | 50.4 | 54.4 |
|  |  | le | 38 | 39.3 | 35.9 | 49.7 | 54.9 | 52.6 | 42.7 |
|  | max | tr | 59.5 | 63.7 | 72.4 | 62.5 | 66.7 | 64.7 | 63.4 |
|  |  | le | 47.6 | 48.6 | 47.8 | 65 | 64.8 | 61.8 | 49.1 |
|  | min | tr | 48 | 48.1 | 54.5 | 37.1 | 47.1 | 41.2 | 44.3 |
|  |  | le | 27.9 | 27.6 | 23 | 39.1 | 40.9 | 42.7 | 31.1 |
|  | comp | tr | 1 vs lev (n.s.) | 2.5 vs lev (***)  2.5 vs 1(*) | 5 vs lev (****)  5 vs 2.5 (****)  5 vs 1 (****) | 1 vs lev (n.s.) | 2.5 vs lev (**)  2.5 vs 1 (n.s.) | 5 vs lev (*)  5 vs 2.5 (n.s.)  5 vs 1 (n.s.) |  |
|  |  | le | 1 vs lev (****) | 2.5 vs lev (****)  2.5 vs 1 (n.s.) | 5 vs lev (****)  5 vs 2.5 (****)  5 vs 1 (*) | 1 vs lev (***) | 2.5 vs lev (****)  2.5 vs 1 (n.s.) | 5 vs lev (****)  5 vs 2.5 (n.s.)  5 vs 1 (n.s.) |  |
| Aperture angle at TD (φ_0_) [°] | n |  | 45 | 75 | 80 | 5 | 60 | 30 | 66 |
|  | mean +/- sd |  | 49.4 +/- 12.8 | 52.1 +/- 8.7 | 56.2 +/-10.4 | 66.4 +/- 1.9 | 35.4 +/-14.9 | 43.7 +/-16.6 | 53.2 +/- 7.3 |
|  | median |  | 49.7 | 51.7 | 56.3 | 66 | 37.7 | 36.5 | 54.4 |
|  | max |  | 69.2 | 64.8 | 74.9 | 69.3 | 62.9 | 80.7 | 64.7 |
|  | min |  | 24.1 | 36.6 | 21.6 | 64.5 | 8.2 | 29.5 | 40.6 |
|  | comp |  | 1 vs lev (n.s) | 2.5 vs lev (n.s.)  2.5 vs 1 (n.s.) | 5 vs lev (n.s.)  5 vs 2.5 (n.s.)  5 vs 1 (n.s.) |  | 2.5 vs lev (****) | 5 vs lev (**)  5 vs 2.5 (*) |  |

n is the number of points used for multiple comparisons. Significance codes: ‘****’ (p < 0.0001); ‘***’ (p < 0.001); ‘**’ (p < 0.01); ‘*’ (p < 0.05); n.s. (non-significant). tr: trailing limb, le: leading limb. TD: touch-down. The aperture angle was computed between trailing and leading limb.

Table S2. Mean, median, max, min values and multiple comparisons for the effective leg during level and step locomotion. For the trailing limb, analyses were performed around TO (TO ± 4%). For the leading limb, at late stance (85% of the stride ± 4%).

|  |  | leg | step up | | | step down | | | level |
| --- | --- | --- | --- | --- | --- | --- | --- | --- | --- |
|  |  |  | 1 cm | 2.5 cm | 5 cm | 1 cm | 2.5 cm | 5 cm |  |
| leg length [m] | n | tr | 81 | 138 | 144 | 29 | 117 | 80 | 198 |
|  |  | le | 83 | 130 | 118 | 5 | 108 | 54 | 252 |
|  | mean +/- sd | tr | 0.103 +/- 0.005 | 0.108 +/- 0.01 | 0.139 +/-0.012 | 0.104 +/- 0.013 | 0.107 +/- 0.005 | 0.08 +/-0.008 | 0.094 +/- 0.005 |
|  |  | le | 0.096 +/- 0.01 | 0.102 +/- 0.006 | 0.108 +/-0.007 | 0.122 +/- 0.001 | 0.11 +/-0.007 | 0.097 +/-0.004 | 0.091 +/- 0.005 |
|  | median | tr | 0.103 | 0.107 | 0.141 | 0.108 | 0.107 | 0.077 | 0.093 |
|  |  | le | 0.1 | 0.104 | 0.109 | 0.122 | 0.111 | 0.095 | 0.091 |
|  | max | tr | 0.111 | 0.126 | 0.155 | 0.123 | 0.117 | 0.096 | 0.107 |
|  |  | le | 0.111 | 0.112 | 0.116 | 0.123 | 0.121 | 0.105 | 0.11 |
|  | min | tr | 0.091 | 0.078 | 0.104 | 0.086 | 0.097 | 0.065 | 0.081 |
|  |  | le | 0.078 | 0.086 | 0.092 | 0.121 | 0.096 | 0.092 | 0.081 |
|  | comp | tr | 1 vs lev (****) | 2.5 vs lev (****)  2.5 vs 1(**) | 5 vs lev (****)  5 vs 2.5 (****)  5 vs 1 (****) | 1 vs lev (***) | 2.5 vs lev (****)  2.5 vs 1 (n.s.) | 5 vs lev (****)  5 vs 2.5 (****)  5 vs 1 (****) |  |
|  |  | le | 1 vs lev (****) | 2.5 vs lev (****)  2.5 vs 1 (***) | 5 vs lev (****)  5 vs 2.5 (****)  5 vs 1 (****) | 1 vs lev (****) | 2.5 vs lev (****)  2.5 vs 1 (**) | 5 vs lev (****)  5 vs 2.5 (****)  5 vs 1 (****) |  |
| Leg angle (α) [°] | n | tr | 81 | 129 | 144 | 36 | 133 | 80 | 198 |
|  |  | le | 83 | 121 | 118 | 18 | 126 | 54 | 252 |
|  | mean +/- sd | tr | 89.1 +/- 10.5 | 96.3 +/-11.8 | 100.5 +/-5.7 | 103.6 +/-19.5 | 82.4 +/-14.6 | 106.2 +/-15.7 | 108.2 +/- 10.7 |
|  |  | le | 85.7 +/-5.8 | 86.1 +/-8.3 | 84.6 +/-4.4 | 94 +/- 5.3 | 79.2 +/-9.4 | 81.4 +/-5.4 | 88.7 +/- 8.5 |
|  | median | tr | 89.7 | 98.4 | 100.2 | 106.1 | 79.8 | 107 | 110.9 |
|  |  | le | 86.4 | 86.8 | 84.6 | 95.8 | 79.2 | 81.2 | 90.1 |
|  | max | tr | 105.7 | 120.3 | 118.7 | 130.5 | 113.2 | 137.4 | 121.5 |
|  |  | le | 97 | 106.3 | 95.2 | 99.6 | 96.2 | 92.3 | 103.1 |
|  | min | tr | 71.4 | 64.9 | 89.6 | 68 | 52.6 | 62 | 69.7 |
|  |  | le | 73.4 | 71.6 | 75.5 | 82.8 | 64.7 | 71.3 | 59.7 |
|  | comp | tr | 1 vs lev (****) | 2.5 vs lev (****)  2.5 vs 1(**) | 5 vs lev (****)  5 vs 2.5 (n.s.)  5 vs 1 (****) | 1 vs lev (n.s.) | 2.5 vs lev (****)  2.5 vs 1 (****) | 5 vs lev (n.s.)  5 vs 2.5 (****)  5 vs 1 (n.s.) |  |
|  |  | le | 1 vs lev (**) | 2.5 vs lev (*)  2.5 vs 1 (n.s.) | 5 vs lev (^****^)  5 vs 2.5 (n.s.)  5 vs 1 (n.s.) | 1 vs lev (*) | 2.5 vs lev (****)  2.5 vs 1 (****) | 5 vs lev (****)  5 vs 2.5 (n.s.)  5 vs 1 (****) |  |

n is the number of points used for multiple comparisons. Significance codes: ‘****’ (p < 0.0001); ‘***’ (p < 0.001); ‘**’ (p < 0.01); ‘*’ (p < 0.05); n.s. (non-significant). tr: trailing limb, le: leading limb. TO: toe-off.

Table S3 Mean, median, max, min values and multiple comparisons between joint angles during level and step locomotion. For the trailing limb, analyses were performed at early stance (15% of the stance ± 4%). For the leading limb, around TD (TD ± 4%).

|  |  | leg | step up | | | step down | | | level |
| --- | --- | --- | --- | --- | --- | --- | --- | --- | --- |
|  |  |  | 1 cm | 2.5 cm | 5 cm | 1 cm | 2.5 cm | 5 cm |  |
| knee angle [°] | n | tr | 42 | 44 | 82 | 38 | 107 | 58 | 259 |
|  |  | le | 81 | 135 | 144 | 36 | 135 | 81 | 184 |
|  | mean +/- sd | tr | 85.2 +/- 8.8 | 90.9 +/- 6.3 | 113.1 +/- 10.1 | 93.1 +/- 8 | 103.7 +/- 8.2 | 90.2 +/-7.3 | 98.3 +/- 9.3 |
|  |  | le | 106.5 +/- 7.1 | 112.5 +/- 9.9 | 109.7 +/- 9.8 | 115.4 +/- 12 | 127.8 +/- 7.7 | 131.5 +/- 7.4 | 120.4 +/- 7.4 |
|  | median | tr | 88 | 91.2 | 112.8 | 91.4 | 104.4 | 89.9 | 97.2 |
|  |  | le | 106.8 | 115.7 | 111.6 | 111.1 | 129.1 | 133.4 | 120.8 |
|  | max | tr | 97.9 | 107.4 | 140.8 | 107.5 | 122.1 | 102.7 | 119.1 |
|  |  | le | 120.7 | 130.3 | 124 | 141.2 | 145.4 | 143.9 | 135.2 |
|  | min | tr | 65.1 | 76.2 | 90.5 | 79.8 | 75.3 | 77 | 80 |
|  |  | le | 91.4 | 90.8 | 77.5 | 97 | 108.3 | 111.4 | 97 |
|  | comp | tr | 1 vs lev (****) | 2.5 vs lev (****)  2.5 vs 1 (n.s.) | 5 vs lev (****)  5 vs 2.5 (****)  5 vs 1 (****) | 1 vs lev (**) | 2.5 vs lev: (****)  2.5 vs 1 (****) | 5 vs lev (****)  5 vs 2.5 (****)  5 vs 1 (n.s.) |  |
|  |  | le | 1 vs lev (****) | 2.5 vs lev (*****)  2.5 vs 1 (***) | 5 vs lev (****)  5 vs 2.5 (n.s.)  5 vs 1 (n.s.) | 1 vs lev (n.s.) | 2.5 vs lev (****)  2.5 vs 1 (****) | 5 vs lev (****)  5 vs 2.5 (*)  5 vs 1 (****) |  |
| INT angle [°] | n | tr | 42 | 44 | 81 | 38 | 107 | 58 | 259 |
|  |  | le | 81 | 135 | 144 | 36 | 135 | 81 | 161 |
|  | mean +/- sd | tr | 99 +/- 10.5 | 114.1 +/-14.5 | 139.3 +/- 9.4 | 110.5 +/- 6.9 | 126.2 +/-12.7 | 94.8 +/-9.3 | 112 +/- 8.6 |
|  |  | le | 111.7 +/- 10 | 114 +/- 10.8 | 121.2 +/- 16.7 | 126.2 +/- 13.9 | 146.4 +/- 12.5 | 148.5 +/- 11.2 | 125.2 +/- 13.5 |
|  | median | tr | 102.2 | 111.6 | 139.7 | 112.7 | 128.9 | 93.8 | 110.4 |
|  |  | le | 109.2 | 115.3 | 124.1 | 123 | 145.7 | 150.7 | 124.8 |
|  | max | tr | 111.6 | 138.2 | 156.1 | 121.2 | 145.5 | 117.4 | 135.7 |
|  |  | le | 138.8 | 132.6 | 145.4 | 152.7 | 171.3 | 164.6 | 154.6 |
|  | min | tr | 79.1 | 87.4 | 123.8 | 100.7 | 94.6 | 82.4 | 95.8 |
|  |  | le | 93.2 | 83.3 | 59.1 | 101.8 | 120.7 | 120.1 | 95.8 |
|  | comp | tr | 1 vs lev (****) | 2.5 vs lev (n.s.)  2.5 vs 1 (***) | 5 vs lev (****)  5 vs 2.5 (****)  5 vs 1 (****) | 1 vs lev (n.s) | 2.5 vs lev (****)  2.5 vs 1 (****) | 5 vs lev (****)  5 vs 2.5 (****)  5 vs 1 (***) |  |
|  |  | le | 1 vs lev (****) | 2.5 vs lev (****)  2.5 vs 1 (n.s.) | 5 vs lev (n.s.)  5 vs 2.5 (**)  5 vs 1 (***) | 1 vs lev (n.s.) | 2.5 vs lev (****)  2.5 vs 1 (****) | 5 vs lev (****)  5 vs 2.5 (n.s.)  5 vs 1 (****) |  |
| TMT angle [°] | n | tr | 42 | 44 | 81 | 38 | 107 | 58 | 249 |
|  |  | le | 81 | 129 | 144 | 27 | 126 | 81 | 135 |
|  | mean +/- sd | tr | 139.6 +/- 5.6 | 134.8 +/-16.5 | 115.5 +/- 14.8 | 129.2 +/- 32 | 130.2 +/-11.7 | 147.5 +/-13.8 | 142.6 +/- 7.4 |
|  |  | le | 159.1 +/- 20.2 | 167.2 +/- 7.2 | 161 +/- 10.1 | 151 +/- 21.3 | 132.9 +/- 14.2 | 133.8 +/- 7 | 158.1 +/- 9.5 |
|  | median | tr | 138.3 | 135.5 | 118.5 | 134.5 | 125.9 | 148.8 | 142.5 |
|  |  | le | 165.2 | 168.2 | 162.9 | 158.7 | 133.2 | 133.7 | 158.5 |
|  | max | tr | 152.2 | 162.7 | 134.3 | 162.3 | 158.9 | 165.2 | 156 |
|  |  | le | 178 | 179.2 | 178.6 | 176.4 | 164 | 155.7 | 177.1 |
|  | min | tr | 128.8 | 106.2 | 73.8 | 13.7 | 114.4 | 109.2 | 124.1 |
|  |  | le | 99.1 | 149.7 | 131.9 | 117.5 | 106.4 | 119.1 | 136.1 |
|  | comp | tr | 1 vs lev (n.s.) | 2.5 vs lev (**)  2.5 vs 1 (n.s.) | 5 vs lev (****)  5 vs 2.5 (****)  5 vs 1 (****) | 1 vs lev (**) | 2.5 vs lev (****)  2.5 vs 1 (n.s.) | 5 vs lev (*)  5 vs 2.5 (****)  5 vs 1 (****) |  |
|  |  | le | 1 vs lev (n.s.) | 2.5 vs lev (****)  2.5 vs 1 (**) | 5 vs lev (*)  5 vs 2.5 (*)  5 vs 1 (n.s.) | 1 vs lev (n.s.) | 2.5 vs lev (****)  2.5 vs 1 (****) | 5 vs lev (****)  5 vs 2.5 (n.s.)  5 vs 1 (****) |  |

n is the number of points used for multiple comparisons. Significance codes: ‘****’ (p < 0.0001); ‘***’ (p < 0.001); ‘**’ (p < 0.01); ‘*’ (p < 0.05); n.s. (non-significant). tr: trailing limb, le: leading limb. TD: touch-down.

Table S4 Mean, median, max, min values and multiple comparisons between joint angles during level and step locomotion. For the leading limb, analyses were performed at late stance (85% of the stance ± 4%). For the trailing limb, around TO (TO ± 4%).

|  |  | leg | step up | | | step down | | | level |
| --- | --- | --- | --- | --- | --- | --- | --- | --- | --- |
|  |  |  | 1 cm | 2.5 cm | 5 cm | 1 cm | 2.5 cm | 5 cm |  |
| knee angle [°] | n | tr | 81 | 130 | 144 | 44 | 135 | 81 | 201 |
|  |  | le | 41 | 69 | 116 | 16 | 113 | 54 | 263 |
|  | mean +/- sd | tr | 64.2 +/- 12.6 | 73.3 +/-13 | 103.4 +/-19.7 | 68.1 +/-16.4 | 63.3 +/-10.4 | 47.9 +/-8.2 | 60.3 +/- 9.8 |
|  |  | le | 53.7 +/- 8.4 | 73.6 +/- 11 | 82 +/- 7.8 | 69.8 +/- 8.7 | 80.6 +/- 7.6 | 78.6 +/- 5.4 | 73.8 +/- 7.1 |
|  | median | tr | 62.6 | 70.4 | 106.9 | 63.4 | 62.8 | 47.1 | 58.3 |
|  |  | le | 53.7 | 76 | 84.7 | 65.9 | 79.4 | 78.9 | 72.8 |
|  | max | tr | 88.2 | 125.4 | 137.7 | 101.6 | 91.2 | 72.8 | 83.1 |
|  |  | le | 69 | 89.3 | 94.2 | 91.1 | 99.8 | 88.5 | 95.5 |
|  | min | tr | 39.4 | 53.5 | 55.7 | 47.8 | 46 | 36.1 | 42.2 |
|  |  | le | 40.6 | 39.3 | 63.5 | 61.7 | 66.4 | 70.3 | 56.3 |
|  | comp | tr | 1 vs lev (*) | 2.5 vs lev (****)  2.5 vs 1(***) | 5 vs lev (****)  5 vs 2.5 (****)  5 vs 1 (****) | 1 vs lev (**) | 2.5 vs lev (*)  2.5 vs 1 (****) | 5 vs lev (****)  5 vs 2.5 (****)  5 vs 1 (****) |  |
|  |  | le | 1 vs lev (****) | 2.5 vs lev (n.s)  2.5 vs 1 (****) | 5 vs lev (****)  5 vs 2.5 (***)  5 vs 1 (****) | 1 vs lev (n.s) | 2.5 vs lev (****)  2.5 vs 1 (n.s.) | 5 vs lev (****)  5 vs 2.5 (n.s.)  5 vs 1 (n.s.) |  |
| INT angle [°] | n | tr | 72 | 130 | 135 | 40 | 135 | 81 | 202 |
|  |  | le | 35 | 69 | 116 | 16 | 113 | 54 | 263 |
|  | mean +/- sd | tr | 80.6 +/- 23.2 | 112.4 +/- 25.7 | 143.3 +/-19.2 | 107.7 +/-27.5 | 105 +/- 28.5 | 76.2 +/- 25.9 | 112.1 +/- 21.6 |
|  |  | le | 92.8 +/- 18.2 | 110.1 +/- 27.4 | 136.2 +/- 13 | 115.5 +/- 6.4 | 130.6 +/- 22.3 | 135.1 +/- 11.7 | 135.2 +/- 14.2 |
|  | median | tr | 82.2 | 112.3 | 145.7 | 107.6 | 104.1 | 74.5 | 114.8 |
|  |  | le | 105.4 | 119.6 | 137.5 | 116.1 | 136.2 | 132.5 | 135.3 |
|  | max | tr | 137.4 | 173.2 | 173.1 | 150.7 | 157.4 | 129.3 | 160.9 |
|  |  | le | 112.9 | 142.8 | 161.5 | 127.9 | 163.4 | 157 | 158.2 |
|  | min | tr | 50.8 | 64.7 | 90.4 | 57.2 | 53.3 | 42 | 65.2 |
|  |  | le | 65.6 | 56.6 | 101.3 | 103.1 | 79.9 | 115.9 | 100.8 |
|  | comp | tr | 1 vs lev (****) | 2.5 vs lev (n.s.)  (2.5 vs 1)  (****) | 5 vs lev (****)  5 vs 2.5  (****)  5 vs 1 (****) | 1 vs lev (n.s) | 2.5 vs lev (*)  2.5 vs 1 (n.s.) | 5 vs lev (****)  5 vs 2.5 (****)  5 vs 1(****) |  |
|  |  | le | 1 vs lev (****) | 2.5 vs lev (****)  2.5 vs 1 (****) | 5 vs lev (n.s.)  5 vs 2.5 (****)  5 vs 1 (****) | 1 vs lev (****) | 2.5 vs lev (n.s.)  2.5 vs 1 (n.s.) | 5 vs lev (n.s.)  5 vs 2.5 (n.s.)  5 vs 1 (n.s.) |  |
| TMP angle [°] | n | tr | 72 | 124 | 135 | 44 | 133 | 80 | 208 |
|  |  | le | 45 | 82 | 143 | 18 | 126 | 58 | 248 |
|  | mean +/- sd | tr | 140.2 +/-21.4 | 131 +/- 29.9 | 142.6 +/-22.6 | 130.3 +/-27.1 | 129.7 +/-25.8 | 120.1 +/-29.1 | 141.6 +/- 21.9 |
|  |  | le | 111.4 +/- 8.8 | 111.1 +/- 25.8 | 95.9 +/- 5.7 | 129.4 +/- 16.5 | 98.9 +/- 27.4 | 91.8 +/- 5.6 | 99.9 +/- 11 |
|  | median | tr | 136 | 133.2 | 142.2 | 131.2 | 130.1 | 117.7 | 143.3 |
|  |  | le | 109.4 | 102.6 | 96.6 | 130.1 | 91.8 | 90.5 | 97.7 |
|  | max | tr | 175.8 | 175.9 | 179.1 | 176 | 172.9 | 169 | 176.6 |
|  |  | le | 127.1 | 165.7 | 112.3 | 159.6 | 164.6 | 107.4 | 136.8 |
|  | min | tr | 79.7 | 55.9 | 96.3 | 87.4 | 80.3 | 73.1 | 93.9 |
|  |  | le | 97.4 | 77.2 | 79.7 | 102 | 60.2 | 84.3 | 82.4 |
|  | comp | tr | 1 vs lev (n.s.) | 2.5 vs lev (**)  2.5 vs 1 (n.s.) | 5 vs lev (n.s.)  5 vs 2.5 (**)  5 vs 1 (n.s.) | 1 vs lev (*) | 2.5 vs lev (****)  2.5 vs 1 (n.s.) | 5 vs lev (****)  5 vs 2.5 (n.s.)  5 vs 1 (n.s.) |  |
|  |  | le | 1 vs lev (****) | 2.5 vs lev (***)  2.5 vs 1 (n.s.) | 5 vs lev (***)  5 vs 2.5 (****)  5 vs 1 (***) | 1 vs lev (****) | 2.5 vs lev (n.s.)  2.5 vs 1 (****) | 5 vs lev (****)  5 vs 2.5 (n.s.)  5 vs 1 (****) |  |

n is the number of points used for multiple comparisons. Significance codes: ‘****’ (p < 0.0001); ‘***’ (p < 0.001); ‘**’ (p < 0.01); ‘*’ (p < 0.05); n.s. (non-significant). tr: trailing limb, le: leading limb. TO: toe-off.

Table S5. Mean, median, max, min values and multiple comparisons between hip cardan angles during level and step locomotion. For the trailing limb, analyses were performed at early stance (15% of the stance ± 4%). For the leading limb, around TD (TD ± 4%).

|  |  | leg | step up | | | step down | | | level |
| --- | --- | --- | --- | --- | --- | --- | --- | --- | --- |
|  |  |  | 1 cm | 2.5 cm | 5 cm | 1 cm | 2.5 cm | 5 cm |  |
| Pro-Re (β) [°] | n | tr | 42 | 44 | 77 | 32 | 107 | 58 | 258 |
|  |  | le | 81 | 126 | 144 | 36 | 135 | 81 | 161 |
|  | mean +/- sd | tr | 46.1 +/- 9 | 48.6 +/- 8.3 | 62.1 +/-11.5 | 43.1 +/-4.1 | 45.8 +/-8.4 | 35.6 +/-4.7 | 41.4 +/- 9.2 |
|  |  | le | 37.3 +/- 8.2 | 41.6 +/- 7.5 | 37.9 +/- 7.5 | 44.4 +/- 8.6 | 51.9 +/- 8.2 | 47.4 +/- 4 | 42.4 +/- 8 |
|  | median | tr | 49.6 | 48.5 | 59 | 41.7 | 45.4 | 34.2 | 41.2 |
|  |  | le | 39.5 | 42.6 | 37.6 | 44.9 | 51.1 | 48.3 | 42.3 |
|  | max | tr | 59.6 | 64 | 89.5 | 50.2 | 63.7 | 47 | 64.2 |
|  |  | le | 50.7 | 60.7 | 53.7 | 63.7 | 71.7 | 53.3 | 63.9 |
|  | min | tr | 32 | 33.8 | 46 | 36.6 | 29.3 | 30 | 22.3 |
|  |  | le | 22.7 | 27.9 | 19.8 | 29.9 | 38.8 | 36.5 | 29.5 |
|  | comp | tr | 1 vs lev (**) | 2.5 vs lev (****)  2.5 vs 1(n.s.) | 5 vs lev (****)  5 vs 2.5 (****)  5 vs 1 (****) | 1 vs lev (n.s.) | 2.5 vs lev (***)  2.5 vs 1(n.s.) | 5 vs lev (****)  5 vs 2.5 (****)  5 vs 1 (***) |  |
|  |  | le | 1 vs lev (****) | 2.5 vs lev (n.s.)  2.5 vs 1 (***) | 5 vs lev (****)  5 vs 2.5 (***)  5 vs 1 (n.s.) | 1 vs lev (n.s.) | 2.5 vs lev (****)  2.5 vs 1 ** | 5 vs lev (****)  5 vs 2.5 (***)  5 vs 1 (n.s.) |  |
| Me-La (α) [°] | n | tr | 42 | 44 | 77 | 32 | 107 | 58 | 258 |
|  |  | le | 81 | 126 | 144 | 36 | 135 | 81 | 161 |
|  | mean +/- sd | tr | -3.8 +/-4.2 | -2.2 +/- 4.5 | 4.5 +/- 4.5 | -6.5 +/- 4.9 | -2.1 +/- 3.1 | -11.2 +/- 2.5 | -6.3 +/- 8.7 |
|  |  | le | -7 +/- 4 | -7.9 +/- 3.7 | -9.3 +/- 5 | -8.5 +/- 6.8 | 2.9 +/- 5.6 | -1 +/- 6.3 | -15 +/- 8.2 |
|  | median | tr | -3.5 | -2.1 | 4.3 | -5.8 | -1.9 | -11.1 | -6 |
|  |  | le | -6.7 | -7.7 | -7.7 | -12.5 | 2 | -2.1 | -16.2 |
|  | max | tr | 2.6 | 6.4 | 12.5 | 7.6 | 3 | -6.4 | 13.1 |
|  |  | le | 0 | 0.8 | 1.1 | 2.8 | 17.9 | 9.2 | 7.4 |
|  | min | tr | -11.3 | -8 | -4.6 | -14.9 | -9.5 | -15.8 | -21.9 |
|  |  | le | -12.6 | -17.6 | -23.8 | -15.6 | -7.3 | -14 | -29.2 |
|  | comp | tr | 1 vs lev (n.s.) | 2.5 vs lev (****)  2.5 vs 1(n.s.) | 5 vs lev (****)  5 vs 2.5 (****)  5 vs 1 (****) | 1 vs lev (n.s.) | 2.5 vs lev (****)  2.5 vs 1(**) | 5 vs lev (****)  5 vs 2.5 (****)  5 vs 1 (***) |  |
|  |  | le | 1 vs lev (****) | 2.5 vs lev (****)  2.5 vs 1 (n.s.) | 5 vs lev (****)  5 vs 2.5 (n.s.)  5 vs 1 (*) | 1 vs lev (****) | 2.5 vs lev (****)  2.5 vs 1 (****) | 5 vs lev (****)  5 vs 2.5 (***)  5 vs 1 (***) |  |
| Ab-Ad (γ) [°] | n | tr | 42 | 44 | 77 | 32 | 107 | 58 | 258 |
|  |  | le | 81 | 126 | 144 | 36 | 135 | 81 | 161 |
|  | mean +/- sd | tr | 26.7 +/- 3.1 | 21.1 +/- 5.4 | 20 +/- 5.7 | 15.9 +/- 4.8 | 28.5 +/- 6.6 | 34.6 +/-3.6 | 28.8 +/- 7.7 |
|  |  | le | 25.3 +/- 6 | 29.9 +/- 6.2 | 24.6 +/- 4.5 | 22.9 +/- 8.1 | 34.5 +/- 11.8 | 34 +/- 5.9 | 37 +/- 10 |
|  | median | tr | 27.5 | 22 | 20.5 | 16.9 | 27.6 | 34.8 | 29.6 |
|  |  | le | 27.4 | 29.5 | 24.9 | 22.3 | 34.4 | 32.1 | 38.3 |
|  | max | tr | 33.9 | 31.1 | 30.2 | 21.1 | 47.6 | 40.3 | 46.9 |
|  |  | le | 35.2 | 41.2 | 34.5 | 37.4 | 63.3 | 48.6 | 59 |
|  | min | tr | 21.1 | 10.7 | 3.2 | 0.8 | 16.7 | 25.3 | 10.9 |
|  |  | le | 15.8 | 12.9 | 12.2 | 10.3 | 3.9 | 25.3 | 12.3 |
|  | comp | tr | 1 vs lev (n.s.) | 2.5 vs lev (****)  2.5 vs 1 (***) | 5 vs lev (****)  5 vs 2.5 (n.s.)  5 vs 1 (****) | 1 vs lev (****) | 2.5 vs lev (n.s.)  2.5 vs 1(****) | 5 vs lev (****)  5 vs 2.5 (***)  5 vs 1 (****) |  |
|  |  | le | 1 vs lev (****) | 2.5 vs lev (****)  2.5 vs 1 (****) | 5 vs lev (****)  5 vs 2.5 (****)  5 vs 1 (n.s.) | 1 vs lev (****) | 2.5 vs lev (n.s.)  2.5 vs 1 (****) | 5 vs lev (**)  5 vs 2.5 (n.s.)  5 vs 1 (***) |  |

n is the number of points used for multiple comparisons. Significance codes: ‘****’ (p < 0.0001); ‘***’ (p < 0.001); ‘**’ (p < 0.01); ‘*’ (p < 0.05); n.s. (non-significant). tr: trailing limb, le: leading limb. TD: touch-down.

Table S6. Mean, median, max, min values and multiple comparisons between hip cardan angles during level and step locomotion. For the leading limb, analyses were performed at late stance (85% of the stance ± 4%). For the trailing limb, around TO (TO ± 4%).

|  |  | leg | step up | | | step down | | | level |
| --- | --- | --- | --- | --- | --- | --- | --- | --- | --- |
|  |  |  | 1 cm | 2.5 cm | 5 cm | 1 cm | 2.5 cm | 5 cm |  |
| Pro-Re (β) [°] | n | tr | 72 | 130 | 135 | 45 | 135 | 81 | 195 |
|  |  | le | 41 | 61 | 99 | 11 | 113 | 54 | 261 |
|  | mean +/- sd | tr | 72.5 +/-6.7 | 69.2 +/-9.9 | 86.4 +/-11.2 | 67.9 +/- 8.7 | 58 +/- 9 | 52.2 +/-10.5 | 57.2 +/- 7.2 |
|  |  | le | 62.1 +/- 8.5 | 67.8 +/- 8.8 | 66.5 +/- 4.5 | 51.9 +/- 10.9 | 59 +/- 12.6 | 55.7 +/- 3.1 | 56.1 +/- 8.9 |
|  | median | tr | 74.8 | 71.3 | 87.4 | 68.2 | 58.4 | 53.3 | 56 |
|  |  | le | 60.4 | 66 | 66.7 | 47 | 56.3 | 54.9 | 54.5 |
|  | max | tr | 81.3 | 82.6 | 108 | 81.5 | 76.6 | 80 | 84.2 |
|  |  | le | 90.2 | 85 | 75.6 | 75.4 | 101.4 | 65.7 | 85.1 |
|  | min | tr | 52.7 | 41 | 59.1 | 47.6 | 36.9 | 32.2 | 42.9 |
|  |  | le | 49.8 | 52.8 | 51.9 | 46.4 | 41.2 | 50.2 | 34.8 |
|  | comp | tr | 1 vs lev (****) | 2.5 vs lev (****)  2.5 vs 1(n.s.) | 5 vs lev (****)  5 vs 2.5 (****)  5 vs 1 (****) | 1 vs lev (****) | 2.5 vs lev (n.s.)  2.5 vs 1 (****) | 5 vs lev (***)  5 vs 2.5 (***)  5 vs 1 (****) |  |
|  |  | le | 1 vs lev (***) | 2.5 vs lev (****)  2.5 vs 1 (n.s.) | 5 vs lev (****)  5 vs 2.5 (n.s.)  5 vs 1 (n.s.) | 1 vs lev (n.s.) | 2.5 vs lev (n.s.)  2.5 vs 1 (n.s.) | 5 vs lev (n.s.)  5 vs 2.5 (n.s.)  5 vs 1 (n.s.) |  |
| Me-La (α) [°] | n | tr | 72 | 130 | 135 | 45 | 135 | 81 | 195 |
|  |  | le | 41 | 61 | 99 | 11 | 113 | 54 | 261 |
|  | mean +/- sd | tr | 4.7 +/- 2.9 | 4.6 +/- 5.1 | 9.4 +/- 5.2 | 12.2 +/- 5.6 | 2.9 +/- 6.6 | 7.5 +/- 7.1 | 10.9 +/- 9.7 |
|  |  | le | 4.9 +/- 6.2 | 4.9 +/- 6.7 | 3.4 +/- 3.8 | -1.1 +/- 1.3 | 3.1 +/- 7.4 | 10.4 +/- 2.4 | 7.6 +/- 10 |
|  | median | tr | 5.2 | 4.8 | 10 | 10.6 | 4.7 | 9.7 | 12 |
|  |  | le | 4.7 | 5.5 | 3.2 | -1.2 | 2.1 | 10.1 | 10.9 |
|  | max | tr | 10.6 | 17.8 | 21.3 | 21.7 | 15.2 | 16.1 | 24.4 |
|  |  | le | 17.1 | 17.4 | 14.2 | 1.2 | 22.7 | 16.9 | 23.4 |
|  | min | tr | -2.4 | -8.6 | -7 | 2.1 | -13 | -17.4 | -14.4 |
|  |  | le | -3.8 | -6.4 | -4.7 | -3.2 | -13.1 | 7.2 | -11.8 |
|  | comp | tr | 1 vs lev (****) | 2.5 vs lev (****)  2.5 vs 1(n.s.) | 5 vs lev (n.s.)  5 vs 2.5 (****)  5 vs 1 (****) | 1 vs lev (n.s.) | 2.5 vs lev (****)  2.5 vs 1 (****) | 5 vs lev (**)  5 vs 2.5 (***)  5 vs 1 (*) |  |
|  |  | le | 1 vs lev (n.s.) | 2.5 vs lev (n.s.)  2.5 vs 1 (n.s.) | 5 vs lev (****)  5 vs 2.5 (n.s.)  5 vs 1 (n.s.) | 1 vs lev (****) | 2.5 vs lev (****)  2.5 vs 1 (n.s.) | 5 vs lev (***)  5 vs 2.5 (***)  5 vs 1 (****) |  |
| Ab-Ad (γ) [°] | n | tr | 72 | 130 | 135 | 45 | 135 | 81 | 195 |
|  |  | le | 41 | 61 | 99 | 11 | 113 | 54 | 261 |
|  | mean +/- sd | tr | 22.1 +/- 11.6 | 14 +/- 7.7 | 9.8 +/- 3.6 | 14.4 +/- 5.7 | 17.4 +/- 8.6 | 15.7 +/- 7.5 | 18.2 +/- 6.3 |
|  |  | le | 17.3 +/- 3.5 | 20.1 +/- 8.5 | 15.2 +/- 4.2 | 12.5 +/- 3.4 | 20.9 +/- 6 | 19.9 +/- 3.2 | 21.2 +/- 8.4 |
|  | median | tr | 18.4 | 13 | 10.4 | 12.4 | 15.8 | 13.4 | 19.2 |
|  |  | le | 17.8 | 18.6 | 15.1 | 13.2 | 20.9 | 20.9 | 22.1 |
|  | max | tr | 50 | 30.6 | 17.7 | 26.6 | 47.1 | 33.1 | 34.8 |
|  |  | le | 24 | 33.6 | 22.4 | 15.8 | 34 | 24.3 | 45.4 |
|  | min | tr | 6.3 | -2.5 | -0.4 | 4.5 | 3.5 | 3.3 | -0.5 |
|  |  | le | 13.2 | -0.2 | 5.7 | 5.7 | 8.3 | 12.1 | 6 |
|  | comp | tr | 1 vs lev (*) | 2.5 vs lev (****)  2.5 vs 1(****) | 5 vs lev (****)  5 vs 2.5 (***)  5 vs 1 (****) | 1 vs lev (****) | 2.5 vs lev (n.s.)  2.5 vs 1 (*) | 5 vs lev (*)  5 vs 2.5 (n.s.)  5 vs 1 (n.s.) |  |
|  |  | le | 1 vs lev (****) | 2.5 vs lev (n.s.)  2.5 vs 1 (n.s.) | 5 vs lev (****)  5 vs 2.5 (***)  5 vs 1 (n.s.) | 1 vs lev (****) | 2.5 vs lev (n.s.)  2.5 vs 1 (****) | 5 vs lev (n.s.)  5 vs 2.5 (n.s.)  5 vs 1 (***) |  |

n is the number of points used for multiple comparisons. Significance codes: ‘****’ (p < 0.0001); ‘***’ (p < 0.001); ‘**’ (p < 0.01); ‘*’ (p < 0.05); n.s. (non-significant). tr: trailing limb, le: leading limb. TO: toe-off.

Table S7. Mean, median, max, min values and multiple comparisons between pelvic pitch angles during level and step locomotion. For the trailing limb, analyses were performed at early stance (15% of the stance ± 4%) and around TO (TO ± 4%). For the leading limb, around TD (TD ± 4%).

| Pitch (β_p_) [°] |  | leg | step up | | | step down | | | level |
| --- | --- | --- | --- | --- | --- | --- | --- | --- | --- |
|  |  |  | 1 cm | 2.5 cm | 5 cm | 1 cm | 2.5 cm | 5 cm |  |
|  | n | tr 15% | 42 | 45 | 78 | 32 | 107 | 58 | 123 |
|  |  | le TD | 81 | 144 | 144 | 45 | 135 | 81 | 144 |
|  |  | tr TO | 81 | 144 | 144 | 45 | 135 | 81 | 144 |
|  | mean +/- sd | tr 15% | -17.6 +/- 6.1 | -17.6 +/- 4.4 | -21.7 +/- 6.7 | -12.6 +/- 6.9 | -17 +/- 6.1 | -6.4 +/- 4.8 | -9.1 +/- 7.8 |
|  |  | le TD | -23.8 +/- 2.7 | -22.5 +/- 6.6 | -25.5 +/- 6.4 | -14.4 +/- 10.8 | -17.3 +/- 6.9 | -9.3 +/- 5.7 | -13.8 +/- 6.8 |
|  |  | tr TO | -21.3 +/- 3.9 | -18.9 +/- 4.6 | -22.9 +/- 5.6 | -14.8 +/- 9.8 | -16.7 +/- 9.5 | -6.9 +/- 3.3 | -10.7 +/- 8.1 |
|  | median | tr 15% | -18.4 | -16.5 | -18.8 | -8.7 | -16.7 | -5.5 | -8.2 |
|  |  | le TD | -23.4 | -21.3 | -25.6 | -11.3 | -16 | -7.7 | -12.5 |
|  |  | tr TO | -20.9 | -19.1 | -21.7 | -11.7 | -15 | -7.2 | -9.4 |
|  | max | tr 15% | -5.6 | -12.3 | -13.3 | -6.1 | -4.5 | 1.2 | 0 |
|  |  | le TD | -19 | -9.2 | -13 | 1.2 | -2.6 | -1.4 | -3.2 |
|  |  | tr TO | -16.4 | -8.1 | -11.4 | 1.2 | -2.9 | 1.1 | -0.3 |
|  | min | tr 15% | -25.6 | -30.4 | -35 | -30.7 | -32.9 | -17 | -33.8 |
|  |  | le TD | -32.4 | -38.3 | -43.3 | -32.1 | -32 | -22.4 | -35.1 |
|  |  | tr TO | -33.2 | -29.9 | -35.4 | -32.7 | -45.1 | -12.8 | -36.6 |
|  | comp | tr 15% | 1 vs lev (****) | 2.5 vs lev (****)  2.5 vs 1(n.s.) | 5 vs lev (****)  5 vs 2.5 (*)  5 vs 1 (*) | 1 vs lev (*) | 2.5 vs lev (****)  2.5 vs 1 (*) | 5 vs lev (*)  5 vs 2.5 (****)  5 vs 1 (***) |  |
|  |  | le TD | 1 vs lev (****) | 2.5 vs lev (****)  2.5 vs 1(n.s.) | 5 vs lev (****)  5 vs 2.5 (**)  5 vs 1 (n.s.) | 1 vs lev (n.s.) | 2.5 vs lev (**)  2.5 vs 1 (n.s.) | 5 vs lev (**)  5 vs 2.5 (****)  5 vs 1 (**) |  |
|  |  | tr TO | 1 vs lev (****) | 2.5 vs lev (****)  2.5 vs 1 (n.s.) | 5 vs lev (****)  5 vs 2.5 (****)  5 vs 1 (n.s.) | 1 vs lev (*) | 2.5 vs lev (****)  2.5 vs 1 (n.s) | 5 vs lev (****)  5 vs 2.5 (****)  5 vs 1 (****) |  |

n is the number of points used for multiple comparisons. Significance codes: ‘****’ (p < 0.0001); ‘***’ (p < 0.001); ‘**’ (p < 0.01); ‘*’ (p < 0.05); n.s. (non-significant). tr: trailing limb, le: leading limb. TD: touch-down, TO: toe-off.

Table S8. Mean, median, max, min values and multiple comparisons between pelvic roll angles during level and step locomotion. For the trailing limb, analyses were performed at early stance (15% of the stance ± 4%) and around TO (TO ± 4%). For the leading limb, around TD (TD ± 4%).

|  |  | leg | step up | | | step down | | | Level |
| --- | --- | --- | --- | --- | --- | --- | --- | --- | --- |
|  |  |  | 1 cm | 2.5 cm | 5 cm | 1 cm | 2.5 cm | 5 cm |  |
| Roll (α_p_) [°] | n | tr 15% | 42 | 45 | 78 | 32 | 107 | 58 | 123 |
|  |  | le TD | 81 | 144 | 144 | 45 | 135 | 81 | 144 |
|  |  | tr TO | 81 | 144 | 144 | 45 | 135 | 81 | 144 |
|  | mean +/- sd | tr 15% | -1.3 +/- 5.6 | -2.1 +/- 4.1 | -5.3 +/- 4.4 | 0.6 +/- 4.8 | -3.8 +/- 4.4 | -1.9 +/- 3.2 | -3.3 +/- 10.7 |
|  |  | le TD | 5.5 +/- 4.6 | 4.9 +/- 4.6 | -0.4 +/- 4.8 | 2.6 +/- 5.1 | 9 +/- 2.9 | 9.2 +/- 3.2 | 2.8 +/- 9.7 |
|  |  | tr TO | 5.7 +/- 4.4 | 3.3 +/- 3.9 | -0.5 +/- 3.9 | 2.1 +/- 5.3 | 8.4 +/- 3.6 | 8.1 +/- 4 | 1.9 +/- 9.1 |
|  | median | tr 15% | -1.3 | -1.8 | -5.3 | 1 | -4.5 | -0.9 | 1.5 |
|  |  | le TD | 6.2 | 5.2 | 0.3 | 4.4 | 9.6 | 10.1 | 4.4 |
|  |  | tr TO | 6.6 | 2.9 | 0 | 4.1 | 8.6 | 9.4 | 5.1 |
|  | max | tr 15% | 6.2 | 5.4 | 4.6 | 6.3 | 6.4 | 2.8 | 11.6 |
|  |  | le TD | 10.9 | 11.2 | 8.2 | 7.4 | 15.4 | 13.9 | 19.2 |
|  |  | tr TO | 11 | 11.7 | 8.2 | 6.9 | 15.4 | 15.8 | 17.7 |
|  | min | tr 15% | -8.4 | -8.3 | -14.5 | -8.3 | -10 | -10.1 | -22.3 |
|  |  | le TD | -6.9 | -8.8 | -12.8 | -8.8 | 2.1 | 2 | -16.5 |
|  |  | tr TO | -8.4 | -8.3 | -14.5 | -8.3 | -10 | -10.1 | -22.3 |
|  | comp | tr 15% | 1 vs lev (n.s.) | 2.5 vs lev (n.s.)  2.5 vs 1(n.s.) | 5 vs lev (n.s.)  5 vs 2.5 (**)  5 vs 1 (****) | 1 vs lev (n.s.) | 2.5 vs lev (n.s.)  2.5 vs 1 (****) | 5 vs lev (n.s.)  5 vs 2.5 (n.s.)  5 vs 1 (n.s.) |  |
|  |  | le TD | 1 vs lev (*) | 2.5 vs lev (*)  2.5 vs 1(n.s.) | 5 vs lev (**)  5 vs 2.5 (****)  5 vs 1 (****) | 1 vs lev (n.s.) | 2.5 vs lev (****)  2.5 vs 1 (****) | 5 vs lev (****)  5 vs 2.5 (n.s.)  5 vs 1 (****) |  |
|  |  | tr TO | 1 vs lev (****) | 2.5 vs lev (n.s.)  2.5 vs 1 (***) | 5 vs lev (**)  5 vs 2.5 (****)  5 vs 1 (****) | 1 vs lev (n.s.) | 2.5 vs lev (****)  2.5 vs 1 (****) | 5 vs lev (****)  5 vs 2.5 (n.s.)  5 vs 1 (****) |  |

n is the number of points used for multiple comparisons. Significance codes: ‘****’ (p < 0.0001); ‘***’ (p < 0.001); ‘**’ (p < 0.01); ‘*’ (p < 0.05); n.s. (non-significant). tr: trailing limb, le: leading limb. TD: touch-down, TO: toe-off.

Table S9. Mean, median, max, min values and multiple comparisons between pelvic yaw angles during level and step locomotion. For the trailing limb, analyses were performed at early stance (15% of the stance ± 4%) and around TO (TO ± 4%). For the leading limb, around TD (TD ± 4%).

|  |  | leg | step up | | | step down | | | Level |
| --- | --- | --- | --- | --- | --- | --- | --- | --- | --- |
|  |  |  | 1 cm | 2.5 cm | 5 cm | 1 cm | 2.5 cm | 5 cm |  |
| Yaw (γ_p_) [°] | n | tr 15% | 42 | 45 | 78 | 32 | 107 | 58 | 123 |
|  |  | le TD | 81 | 144 | 144 | 45 | 135 | 81 | 144 |
|  |  | tr TO | 81 | 144 | 144 | 45 | 135 | 81 | 144 |
|  | mean +/- sd | tr 15% | 0.7 +/- 5.1 | 0.7 +/- 6 | -4.9 +/- 4 | 3.5 +/- 3.6 | -2.4 +/- 7.7 | -3.1 +/- 4.1 | 1 +/- 5.3 |
|  |  | le TD | 0.6 +/- 3.2 | 1.7 +/- 7 | 0.6 +/- 5.8 | 0 +/- 4.8 | 8.7 +/- 7.1 | 7.9 +/- 4.8 | -0.2 +/- 3.7 |
|  |  | tr TO | 1.1 +/- 3.5 | 1 +/- 5.4 | -0.8 +/- 5.8 | 0.2 +/- 3.4 | 8.2 +/- 7.8 | 6.7 +/- 5 | -0.2 +/- 5.1 |
|  | median | tr 15% | 2.9 | 2.1 | -5.3 | 4.1 | -2.6 | -3.1 | 0.6 |
|  |  | le TD | -0.1 | 2.7 | 0 | 1.7 | 7.6 | 8 | -0.8 |
|  |  | tr TO | 0.2 | 1.2 | -0.3 | 0.2 | 9.1 | 7.9 | 0.6 |
|  | max | tr 15% | 7.6 | 7.6 | 5.2 | 12.6 | 15.4 | 4 | 15.1 |
|  |  | le TD | 6.8 | 16.2 | 13.3 | 6.5 | 22.8 | 15.6 | 7.5 |
|  |  | tr TO | 8.1 | 15.3 | 10.3 | 4.3 | 22.8 | 14.3 | 10.5 |
|  | min | tr 15% | -10 | -11.3 | -12.3 | -3.8 | -23.7 | -11.4 | -9.4 |
|  |  | le TD | -3.9 | -10.2 | -14.3 | -12.1 | -4.1 | -1.3 | -5.8 |
|  |  | tr TO | -4.7 | -10.3 | -15.6 | -10 | -5.6 | -3.8 | -12 |
|  | comp | tr 15% | 1 vs lev (n.s.) | 2.5 vs lev (n.s.)  2.5 vs 1(n.s.) | 5 vs lev (****)  5 vs 2.5 (****)  5 vs 1 (****) | 1 vs lev (*) | 2.5 vs lev (***)  2.5 vs 1 (****) | 5 vs lev (****)  5 vs 2.5 (n.s.)  5 vs 1 (****) |  |
|  |  | le TD | 1 vs lev (n.s.) | 2.5 vs lev (*)  2.5 vs 1(n.s.) | 5 vs lev (n.s.)  5 vs 2.5 (n.s.)  5 vs 1 (n.s.) | 1 vs lev (n.s.) | 2.5 vs lev (****)  2.5 vs 1 (****) | 5 vs lev (****)  5 vs 2.5 (n.s.)  5 vs 1 (****) |  |
|  |  | tr TO | 1 vs lev (*) | 2.5 vs lev (*)  2.5 vs 1 (n.s.) | 5 vs lev (n.s.)  5 vs 2.5 (n.s.)  5 vs 1 (n.s) | 1 vs lev (n.s.) | 2.5 vs lev (****)  2.5 vs 1 (****) | 5 vs lev (****)  5 vs 2.5 (n.s.)  5 vs 1 (****) |  |

n is the number of points used for multiple comparisons. Significance codes: ‘****’ (p < 0.0001); ‘***’ (p < 0.001); ‘**’ (p < 0.01); ‘*’ (p < 0.05); n.s. (non-significant). tr: trailing limb, le: leading limb. TD: touch-down, TO: toe-off.

Table S1b. Mean, median, max, min values and multiple comparisons for the effective leg during level and step locomotion. For the trailing limb, analyses were performed at early stance (15% of the stride ± 4%). For the leading limb, around TD (TD ± 4%).

|  |  | leg | step up | | | step down | | | level |
| --- | --- | --- | --- | --- | --- | --- | --- | --- | --- |
|  |  |  | 1 cm | 2.5 cm | 5 cm | 1 cm | 2.5 cm | 5 cm |  |
| leg length  velocity ($\dot{l}$)  [m s^-1^]  For the leading limb  ($\dot{l}$_0_) | n | tr | 48 | 62 | 79 | 10 | 89 | 57 | 248 |
|  |  | le | 90 | 137 | 144 | 18 | 108 | 81 | 131 |
|  | mean +/- sd | tr | -0.337 +/- 0.156 | -0.153 +/- 0.353 | -0.228 +/- 0.113 | -0.464 +/- 0.389 | -0.212 +/- 0.353 | -0.245 +/- 0.123 | -0.373 +/- 0.105 |
|  |  | le | -0.327 +/- 0.316 | -0.042 +/- 0.513 | -0.002 +/- 0.435 | -0.257 +/- 0.532 | -0.334 +/- 0.299 | -0.455 +/- 0.174 | -0.079 +/- 0.506 |
|  | median | tr | -0.357 | -0.125 | -0.189 | -0.49 | -0.226 | -0.211 | -0.376 |
|  |  | le | -0.351 | -0.162 | -0.103 | -0.235 | -0.373 | -0.439 | -0.296 |
|  | max | tr | 0.001 | 0.935 | -0.05 | 0.069 | 0.913 | -0.031 | -0.165 |
|  |  | le | 0.658 | 2.341 | 1.239 | 0.574 | 0.772 | 0.039 | 1.262 |
|  | min | tr | -0.69 | -1.015 | -0.53 | -0.998 | -1.971 | -0.539 | -0.674 |
|  |  | le | -0.979 | -0.945 | -0.683 | -1.162 | -0.961 | -0.914 | -0.746 |
|  | comp | tr | 1 vs lev (n.s.) | 2.5 vs lev (****)  2.5 vs 1(n.s) | 5 vs lev (****)  5 vs 2.5 (n.s.)  5 vs 1 (n.s.) | 1 vs lev (n.s.) | 2.5 vs lev (***)  2.5 vs 1 (n.s.) | 5 vs lev (****)  5 vs 2.5 (n.s.)  5 vs 1 (n.s.) |  |
|  |  | le | 1 vs lev (****) | 2.5 vs lev (n.s.)  2.5 vs 1 (n.s.) | 5 vs lev (n.s)  5 vs 2.5 (****)  5 vs 1 (****) | 1 vs lev (n.s.) | 2.5 vs lev (****)  2.5 vs 1 (n.s.) | 5 vs lev (****)  5 vs 2.5 (n.s.)  5 vs 1 (n.s.) |  |
| Leg angular velocity ($\dot{\alpha}$) [° s^-1^]  For the leading limb  ($\dot{\alpha}$_0_) | n | tr | 48 | 52 | 79 | 24 | 107 | 57 | 248 |
|  |  | le | 90 | 128 | 144 | 18 | 126 | 81 | 131 |
|  | mean +/- sd | tr | 207.7 +/- 89.6 | 101.6 +/- 223.4 | 231.2 +/- 85.1 | 184 +/- 168.1 | 131.4 +/- 161.9 | 123.4 +/- 81.4 | 254.3 +/- 69.9 |
|  |  | le | 256.4 +/- 91.2 | 235.1 +/- 149.4 | 319 +/- 222.7 | 363.5 +/- 134.8 | 243.4 +/- 158 | 200.2 +/- 125.8 | 301.7 +/- 142.3 |
|  | median | tr | 215.1 | 97.3 | 217.3 | 189.8 | 136.6 | 127.2 | 262.7 |
|  |  | le | 269.8 | 213.2 | 305.9 | 360.8 | 223.3 | 159 | 265.6 |
|  | max | tr | 448.3 | 514.6 | 426.2 | 662.4 | 742.1 | 253.7 | 418.1 |
|  |  | le | 439.6 | 638.2 | 814.6 | 672.2 | 630.2 | 588 | 878.1 |
|  | min | tr | 7 | -846.4 | 101.1 | -109.8 | -511.2 | -62.6 | 107.7 |
|  |  | le | 43.8 | -26.5 | -693.9 | 107.5 | -204.4 | 45 | 85.1 |
|  | comp | tr | 1 vs lev (**) | 2.5 vs lev (****)  2.5 vs 1(*) | 5 vs lev (n.s.)  5 vs 2.5 (***)  5 vs 1 (n.s.) | 1 vs lev (n.s.) | 2.5 vs lev (****)  2.5 vs 1 (n.s.) | 5 vs lev (****)  5 vs 2.5 (n.s.)  5 vs 1 (n.s.) |  |
|  |  | le | 1 vs lev (*) | 2.5 vs lev (***)  2.5 vs 1 (n.s.) | 5 vs lev (n.s.)  5 vs 2.5 (***)  5 vs 1 (n.s.) | 1 vs lev (n.s.) | 2.5 vs lev (**)  2.5 vs 1 (*) | 5 vs lev (****)  5 vs 2.5 (n.s.)  5 vs 1 (***) |  |
| Aperture angle velocity at TD ($\dot{\emptyset_{0}}$)  [° s^-1^] | n |  | 45 | 75 | 80 | 5 | 60 | 30 | 65 |
|  | mean +/- sd |  | -92.4 +/-372.9 | -30.9 +/-283.9 | 41.8 +/-356.5 | 622.1 +/-196.4 | -272.9 +/-307.1 | 212.2 +/-52 | 133.8 +/- 117.9 |
|  | median |  | -113.4 | -29.2 | -14.9 | 538.9 | -265.9 | 201.9 | 154.4 |
|  | max |  | 692.7 | 829.2 | 1478.3 | 861.1 | 496.5 | 295 | 376.1 |
|  | min |  | -982.8 | -787.2 | -353.4 | 402.5 | -854.4 | 144.2 | -89.3 |
|  | comp |  | 1 vs lev (n.s) | 2.5 vs lev (n.s.)  2.5 vs 1 (n.s.) | 5 vs lev (n.s.)  5 vs 2.5 (n.s.)  5 vs 1 (n.s.) |  | 2.5 vs lev (****) | 5 vs lev (**)  5 vs 2.5 (*) |  |

n is the number of points used for multiple comparisons. Significance codes: ‘****’ (p < 0.0001); ‘***’ (p < 0.001); ‘**’ (p < 0.01); ‘*’ (p < 0.05); n.s. (non-significant). tr: trailing limb, le: leading limb. TD: touch-down. The aperture angle was computed between trailing and leading limb.
